# On the Reduction of Computational Complexity of Deep Convolutional Neural Networks †

**DOI:** 10.3390/e20040305

**Published:** 2018-04-23

**Authors:** Partha Maji, Robert Mullins

**Affiliations:** Department of Computer Science and Technology, University of Cambridge, William Gates Building, 15 JJ Thomson Avenue, Cambridge CB3 0FD, UK

**Keywords:** convolutional neural network, deep learning, computational optimization, hardware implementation

## Abstract

Deep convolutional neural networks (ConvNets), which are at the heart of many new emerging applications, achieve remarkable performance in audio and visual recognition tasks. Unfortunately, achieving accuracy often implies significant computational costs, limiting deployability. In modern ConvNets it is typical for the convolution layers to consume the vast majority of computational resources during inference. This has made the acceleration of these layers an important research area in academia and industry. In this paper, we examine the effects of co-optimizing the internal structures of the convolutional layers and underlying implementation of fundamental convolution operation. We demonstrate that a combination of these methods can have a big impact on the overall speedup of a ConvNet, achieving a ten-fold increase over baseline. We also introduce a new class of fast one-dimensional (1D) convolutions for ConvNets using the Toom–Cook algorithm. We show that our proposed scheme is mathematically well-grounded, robust, and does not require any time-consuming retraining, while still achieving speedups solely from convolutional layers with no loss in baseline accuracy.

## 1. Introduction

Convolutional neural networks (ConvNets) are becoming a mainstream technology for an array of new embedded applications, including speech recognition, language translation, object detection, image recognition, and numerous other complex tasks ([1,2,3,4,5]). This breakthrough has been made possible by recent progress in deep learning, although the theoretical understanding remains, however, unsatisfactory. Basic questions about optimal architecture, the number of required layers, and the number of neurons per layer are not well understood. Most state-of-the-art deep models typically require millions of parameters and billions of operations to produce human-level accuracy ([6,7,8]). The memory and computational requirements in particular complicate the deployment of deep neural networks on low power-embedded platforms as they have a very limited computational and power budget. To avoid running end-to-end inference on embedded systems, the current state-of-the-art solutions enable this type of application by off-loading the computation to cloud-based infrastructures where server-grade machines (GPUs and other application-specific accelerators) perform the heavy number crunching. Unfortunately, the cloud-assisted approach places severe limitations on the usability and scalability of deep learning-based embedded and Internet of Things (IoT) applications. First and foremost, the user data is sent across the cloud, with serious privacy implications. Second, sending lots of data (e.g., every frame of a video) over a wireless network consumes significant power due to the communication overhead. For applications where continuous data exchange is required between the server and the mobile device, latency is also a big concern. For example, a wearable continuous glucose level monitoring sensor must detect an abnormal condition and must perform an action in real time. The third limitation is the scalability, which has mid to long-term implications. Gartner Inc., one of the world’s leading research and advisory companies, estimates that by 2020, 26 billion IoT units will be installed globally [9]. The staggering amount of data generated by IoT devices will easily exceed the storage limits of cloud infrastructure. To truly scale deep learning-based applications globally in various scenarios, we have to enable these applications without the requirement of always having to connect to the cloud infrastructure.

**Figure 1:**
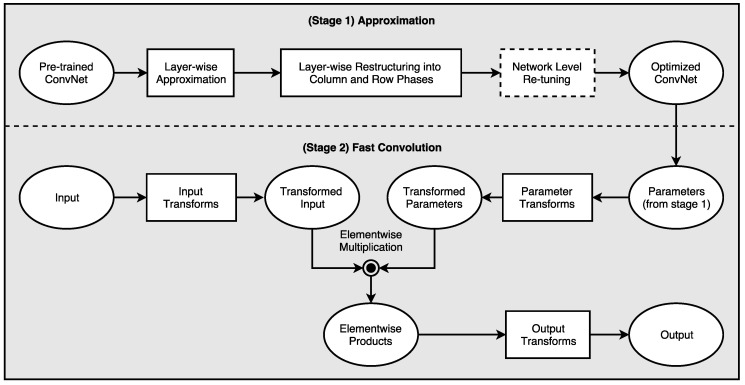
One-Dimensional Fast Approximate Low-rank CONvolution (1D-FALCON): A high-level optimization pipeline consisting of two main stages.

In this paper, we propose a robust and easy-to-implement acceleration scheme, known as One-Dimensional Fast Approximate Low-rank CONvolution (1D-FALCON), which can be applied on readily available state-of-the-art pre-trained models. Very recently, Tishby et al. showed that deep neural networks can be explained from an information-theoretic approach [10]. The author showed us that the goal of deep learning can be expressed as an information-theoretic trade-off between compression and prediction accuracy. Our proposed scheme exploits the inherent redundancy present in the convolution layers in order to reduce the compute complexity of deep networks. Additionally, we decompose each filter bank into multiple one-dimensional (1D) low-rank vectors to reduce the total number of operations required per layer. We then apply a modified version of the Toom–Cook algorithm to compute the convolution using one-dimensional filters to further reduce the number of multiplications in discrete convolution. Figure 1 presents the high-level optimization pipeline from our 1D-FALCON scheme.

Although many earlier studies have focused on reducing overall memory footprint by compression, only a few have aimed at speeding up convolutional layers. Unlike many previously proposed pruning and regularization techniques, our scheme does not involve any time-consuming iterative retraining cycle. Furthermore, since rank selection and decomposition are only dependent on the individual layer’s inherent property, each convolution layer can be approximated in parallel. Our approximation scheme is mathematically well-grounded, robust, and thus easily tunable using numerical formulation, without sacrificing baseline accuracy. To the best of our knowledge, this paper is the first to study a co-optimization scheme that combines both the one-shot low-rank model approximation technique and a fast arithmetic scheme that exploits convolutions by separability.

## 2. Related Work

Model pruning has been used both to reduce over-fitting and the memory footprint. Optimal brain damage [11] and optimal brain surgery [12] are early examples of pruning aimed at reducing the number of connections within a network. Recently, Han et al. proposed a pruning scheme for ConvNets aimed at reducing the total number of parameters in the entire network [7,13]. However, the authors in this paper mentioned that it is challenging to achieve a significant runtime speedup of a convolutional network with conventional direct implementation. In addition, the pruning-based scheme involves a very long iterative pruning and retraining cycle. For example, it took seven days to retrain the pruned five (convolution)-layer AlexNet [7], which is not practical for fast time-to-market products.

Liu et al. [14] proposed a sparse convolutional neural network (SCNN) model that exploits both inter-channel and intra-channel redundancy to maximize sparsity in a model. This method is very effective for parameter reduction in the fully-connected layers. The retraining stage with a modified cost function is very time consuming.

Denton et al. showed in recent research that the generalized eigendecomposition-based truncation can help to reduce parameters from the fully-connected layers [15]. However, the authors did not consider the computation-intensive convolutional layers. Jaderberg et al. proposed a singular value decomposition-based technique for layer-by-layer approximation [16]. Their methodology uses iterative steps where a layer can only be approximated after the previous layer has been compressed. The author used an updated loss function to learn the low-rank filters, which is again a time-consuming process. The author also reported that simultaneous approximation of all the layers in parallel is not efficient. Mamalet et al. designed the model to use low-rank filters from scratch and combine them with the pooling layer [17]. However, their technique cannot be applied to general network design. Sironi et al. showed that learning-based strategies can be used to obtain separable (rank-1) filters from multiple filters, allowing large speedup with minimal loss in accuracy [18]. We build our methodology on this fundamental idea. Instead of learning separable filters, we use a one-shot approach which can be applied statically.

Gupta et al. [19] studied the effect of limited precision data representation in the context of training ConvNets. They observed that ConvNets can be trained using only 16-bit wide fixed-point number representation with little to no degradation in the classification accuracy. A number of optimization schemes have been proposed recently that recommend use of fewer bits to represent the parameters and datapaths [13,20,21]. Our scheme is orthogonal to these techniques and can be combined with quantization to further reduce the computational complexity and storage requirements.

Cong et al. showed that by using Strassen’s algorithm, computation complexity in convolutional layers can be reduced by up to 47% [6]. Vasilache et al. used an FFT-based scheme to speed up convolutions, which are not very effective for small filters [22]. Recently, both nVidia’s cuDNN and Intel’s MKL library added support for Winograd’s algorithm to speed up convolutions, as originally proposed by Lavin et al. [23]. Although combining sparse methods and Winograd’s convolution holds the potential to achieve significant speedup, pruning Winograd kernels to induce sparsity poses challenges [24].

## 3. Optimization of Deep Convolutional Neural Networks—An Information-Theoretic Approach

**Figure 2:**
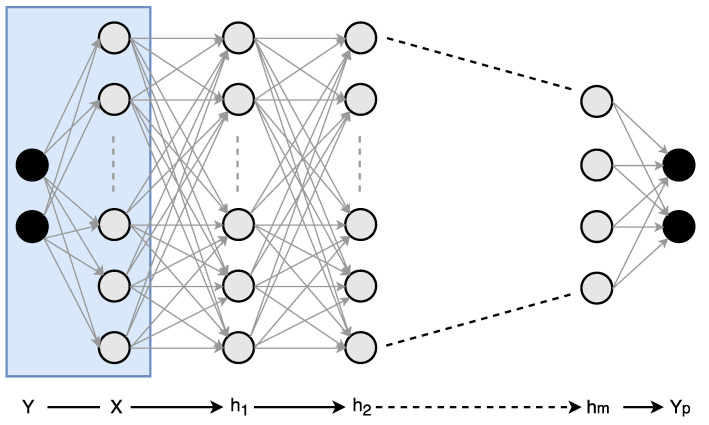
An example of a deep neural network with an input layer *X*, output layer $Y_{p}$, and *m* hidden layers in between. During the training phase, the desired output *Y* is observed and is used to learn the connectivity matrices between the layers. In the inference phase, the network forms a Markov chain, which predicts output $Y_{p}$ for any input *X*.

A typical deep neural network (or deep convolutional neural network) has a huge parameter space, and using the stochastic gradient descent (SGD), one can exponentially arrive at many optimal solutions consisting of different numbers of layers, layer sizes, and numbers of parameters. Although the optimal size of a network (e.g., number of layers, neurons per layer etc.) for a given dataset is unknown at the start, we are able to find a number of alternative approximate networks which yields the same desired accuracy. Tishby et al. recently showed that the organization of deep neural networks can be analyzed using information theory [25]. In their research, the authors demonstrated that an information-theoretic approach can help us to better understand both the learning process and internal representation of deep networks. Typically, in a deep neural network, the input denoted by *X* is a high-dimensional variable, being a low-level representation of data such as pixels of an image, whereas the desired output, *Y*, has a significantly lower dimensionality of the predicted categories. In between the input and the output layer, the structure of deep network forms a Markov chain of intermediate representations made out of many hidden layers—$h_{1},h_{2},\ldots ,h_{m}$ (see Figure 2). In supervised learning we are interested in good representations of the input patterns that enable good predictions of the labels. The deep neural network obtains a Markov chain of such representations, the hidden layers, by minimization of the empirical error over the weights of the network layer by layer. This optimization takes place via stochastic gradient descent (SGD), using a noisy estimate of the gradient of the empirical error of each weight through back propagation. This SGD-based optimization process has two distinct phases: empirical error minimization and representation compression. During the first phase of the SGD-based training process, the network tries to memorize the data using maximum entropy weight distribution. In the second phase of the training, it adds noise to the network, which helps to generalize. How much information flows between the input and the output of a layer defines the trade-off between complexity and accuracy. Mutual information is a measure of correlation between different variables. Using the ReLU activation function the information is also compressed at each layer. In our research we noticed that deep neural networks trained using SGD-based optimization resulted in a lot of correlated filters in hidden layers. We exploit this redundancy to trade off complexity with accuracy. The following section covers this trade-off process in more detail.

## 4. Methodology

The proposed 1D-FALCON scheme consists of two main stages, namely, an approximation stage followed by a fast arithmetic stage, as shown in Figure 1. To achieve this we first approximate each convolutional layer to the necessary level to reduce computational complexity and then decompose each filter bank into two rank-1 filter banks by introducing an intermediate layer in between. If the classification accuracy drops after the layer restructuring stage we fine-tune the model using the training dataset. Then, we apply a modified version of the Toom–Cook algorithm, which computes each 1D convolution for a chosen set of distinct data points, to further reduce the number of strong operations (in this case multiplications). We will show that the combined application of these two schemes results in a significant reduction in computational complexity. In the following few sections describe each phase of our optimization pipeline in detail. We first introduce the idea of a separable filter in the context of convolution.

### 4.1. Separable Filters

The concept of separable filters by splitting convolution operations into convergent sums of matrix-valued stages was proposed by Hummel and Lowe in the 1980s before ConvNet became popular for automatic feature learning [26]. This property was exploited in many early image-processing filters—e.g., the Sobel edge detection filter, the Gaussian blurring filter, etc. This approach is very powerful but restricted to filters that are decomposable, which is often not the case for a trained filter such as in ConvNet. However, due to the presence of inherent redundancy between different filters or feature maps within a layer, this property can be exploited in the acceleration of ConvNet models.

Consider an arbitrary kernel of a ConvNet described by the (m×n) matrix W.

(1) $$W=\left[\begin{array}{cccc} \alpha_{00} & \alpha_{01} & .. & \alpha_{0n}\\ \alpha_{10} & \alpha_{11} & .. & \alpha_{1n}\\ .. & .. & .. & ..\\ \alpha_{m0} & \alpha_{m1} & .. & \alpha_{mn} \end{array}\right]$$

We say that kernel W is separable when it can be split into the outer product of an *m*-length column vector *v* and an *n*-length row vector *h* as follows:

(2) $$W=VH^{T}=\left[\begin{array}{c} v_{0}\\ v_{1}\\ ..\\ v_{m} \end{array}\right]\left[\begin{array}{cccc} h_{0} & h_{1} & .. & h_{n} \end{array}\right]$$

or, W can be explicitly expressed as:

(3) $$W=VH^{T}=\left[\begin{array}{cccc} v_{0}h_{0} & v_{0}h_{1} & .. & v_{0}h_{n}\\ v_{1}h_{0} & v_{1}h_{1} & .. & v_{1}h_{n}\\ .. & .. & .. & ..\\ v_{m}h_{0} & v_{m}h_{1} & .. & v_{m}h_{n} \end{array}\right]$$

From Equations (1) and (3), it is apparent that a separable kernel has equivalent rows and columns. To store the original kernel W in Equation (1), it would require (mn) space. However, if the kernel W is a separable matrix, then we see from Equation (3) that it would require (m+n) space. As *m* and *n* becomes large and original kernel is separable W, one can see that substantial savings in computational time and storage will be achieved.

**Figure 3:**
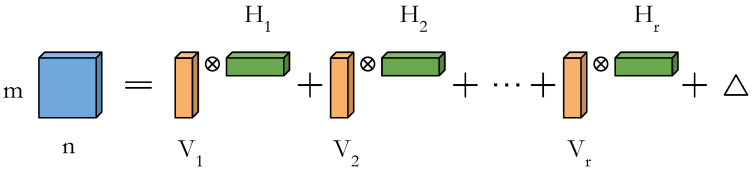
A two-dimensional (2D) matrix can be represented by the sum of *r* rank-1 updates.

Unfortunately, we cannot generally expect that any trained kernel in ConvNet satisfies such stringent conditions. The collection of kernels in a ConvNet is generally of full rank and expensive to convolve with large images. However, we can aim for W to be approximately separable such that

(4) $$W=VH^{T}+E$$

where E is an error kernel, whose importance we would like to be as small as possible in relation to the original kernel W. We can further generalize Equation (4) in the following form:

(5) $$\begin{array}{cc} W & =V_{1}H_{1}^{T}+V_{2}H_{2}^{T}+\ldots +V_{i}H_{i}^{T}+\ldots +V_{r}H_{r}^{T}+E_{r}\\ & =U_{1}+U_{2}+\ldots +U_{i}+\ldots +U_{r}+E_{r} \end{array}$$

where each term,

(6) $$U_{i}=V_{i}H_{i}^{T}$$

is an exactly separable rank-1 outer product of a column vector of length *m* and row vector of length *n*, and $E_{r}$ is the error matrix associated with *r*-term approximation of original kernel W as shown in Figure 3. Eckart and Young showed that the SVD is the solution to the problem of minimizing $E_{r}$ [27]. Furthermore, if the original kernel W can be well approximated by *r* rank-1 updates, we will only require r(m+n) parameters to describe the kernel instead of original mn elements. The key idea here is that if we choose *r* such that r(m+n)<<mn, then it would require less storage and computation. We can extend this idea to the convolutional neural network to reduce the overall cost of computation.

### 4.2. Layerwise Approximation and Convolution by Separability

In ConvNets, multiple layers of convolutional filter (also known as kernel) banks are stacked on top of each other, followed by a non-linear activation function. Significant redundancy exists between those spatial filter dimensions and also along cross-channel feature maps. Most of the previous research has focused on either exploiting approximation along spatial filter dimensions or along one of the feature channel dimensions. In our approach, we aim at approximating the redundancy across both the input and output feature maps.

Let us assume, in a convolutional neural network, that a four-dimensional kernel can be represented as $W\in R^{F_{I}\times \left(m\times n\right)\times F_{O}}$, where spatial two-dimensional kernels are of size (m×n) and $F_{I}$, $F_{O}$ are the input and output channels within a layer, respectively. We can also represent an input feature map as $X\in R^{M\times N\times F_{I}}$ and corresponding kernels as $W_{i}\in R^{m\times n\times F_{I}}$ for *i*th set of weights, where each input feature map is of size (M×N). The original convolution for the *i*th set of weights in a given layer now becomes

(7) $$W_{i}*X=\sum_{f=1}^{F_{I}}W_{i}^{f}*x^{f}$$

Our goal is to find an approximation of kernel $W_{i}$, such that $W_{i}={\tilde{W}}_{i}+E$. Using the concept of separable filters [18], let us assume that for a small error E, the chosen rank is *R*. How the rank *R* is chosen will be explained in the next section. The modified kernel now can be represented by Equation (8), where $V\in R^{R\times (m\times 1\times F_{I})}$ is the approximate column kernel, and $H\in R^{F_{O}\times \left(1\times n\times R\right)}$ is the approximate row kernel.

(8) $$\tilde{W_{i}}*X=\sum_{f=1}^{F_{I}}\sum_{r=1}^{R}H_{i}^{r}{\left(V_{r}^{f}\right)}^{T}*x^{f}=\sum_{r=1}^{R}H_{i}^{r}*\left(\sum_{f=1}^{F_{I}}V_{r}^{f}*x^{f}\right)$$

**Figure 4:**
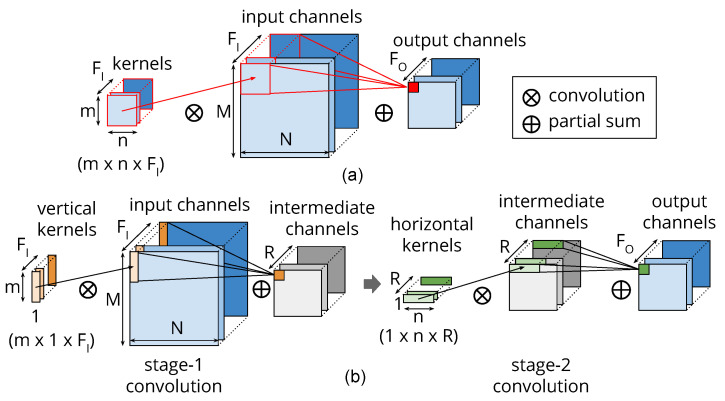
(**a**) The original convolution with a (m×n) kernel. (**b**) The two-stage approximate convolution using a (m×1) column kernel in stage 1 followed by a (1×n) row kernel in stage 2. There are R channels in the intermediate virtual layer.

Figure 4 depicts the idea of re-constructing the convolution layer using the newly constructed column and row low-rank kernels and compares them with the original two-dimensional (2D) direct convolution. We compute the column and row kernels (V,H) statically using generalized eigenvalue decomposition by minimizing the error E. Since we decide the magnitude of the approximation statically, we avoid the long running time of learning-based techniques. Additionally, as the approximation is an inherent property of each layer, we can restructure all the convolutional layers in a ConvNet in parallel, which also saves time. If the accuracy of a model drops at this stage after approximating all the layers, we fine-tune the complete model once using the training dataset.

### 4.3. Rank Search and Layer Restructuring Algorithm

The rank *R* is chosen by the one-shot minimization criterion described before. We apply singular value decomposition on the 2-D tensor $R^{\left(F_{I}m\right)\times \left(nF_{O}\right)}$, which we obtain from the original four-dimensional (4D) tensor $R^{F_{I}\times m\times n\times F_{O}}$. Unlike other minimization criteria such as the Mahalanobis distance metric or the data covariance distance metric [15], our simple criterion gives us an exact decomposition. Algorithm 1 describes the main steps of our low-rank approximation and ConvNet layer restructuring scheme.

**Algorithm 1:** Rank approximation and the layer restructuring algorithm

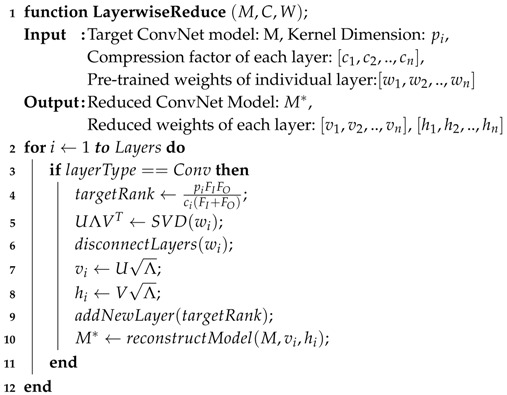



### 4.4. The Modified Toom–Cook’s Fast 1D Convolution

Once we have obtained newly constructed multi-stage 1D convolution layers, we apply a modified version of the Toom–Cook algorithm to further reduce the number of multiplications. In the Toom–Cook method, a linear convolution can be written as product of two polynomials in the real field ([28,29]).

(9) s(p)=w(p)x(p),wheredeg[x(p)]=N−1,deg[w(p)]=L−1

**Figure 5:**
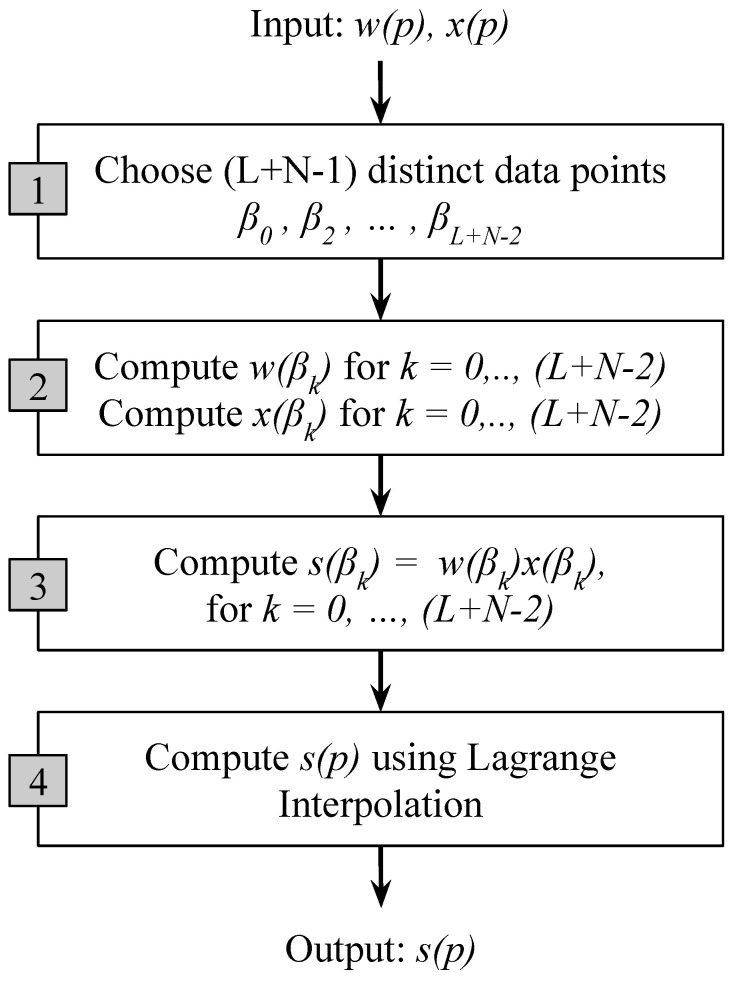
Steps in the modified Toom–Cook algorithm.

The output polynomial s(p) has a degree L+N−2 and L+N−1 different coefficients. Instead of explicitly multiplying the polynomials w(p) and x(p) using the discrete convolution, the Toom–Cook algorithm evaluates the polynomials w(p) and x(p) for a set of data points $\beta_{i}$ and then multiplies their values $s\left(\beta_{i}\right)=w\left(\beta_{i}\right)x\left(\beta_{i}\right)$. Afterwards, the product polynomials s(p) are constructed using the Lagrange interpolation (see Figure 5). The algorithm consists of four steps:

1. Choose L+N−1 distinct data points $\beta_{0}$, $\beta_{1}$,…,$\beta_{L+N-2}$.
2. Evaluate $w(\beta_{i})$ and $x(\beta_{i})$ for all the data points.
3. Compute $s\left(\beta_{i}\right)=w\left(\beta_{i}\right)x\left(\beta_{i}\right)$.
4. Finally, compute s(p) by Lagrange interpolation as follows:
  (10) $$s\left(p\right)=\sum_{i=0}^{L+N-2}s\left(\beta_{i}\right)\frac{\prod_{j\neq i}\left(x-\beta_{j}\right)}{\prod_{j\neq i}\left(\beta_{i}-\beta_{j}\right)}$$

Since (L+N−1) distinct data points are chosen in step 1, a total of (L+N−1) multiplications are required in step 3. The Toom–Cook algorithm can also be viewed as a method of factoring matrices and can be expressed as the following form (⊙ denotes element-wise multiplication):

(11) s(p)=S[{Ww(p)}⊙{Xx(p)}]

where W,X and *S* are the transform matrix for kernels, input, and output, respectively. The cost of computing {Ww(p)} gets amortized over reuse of the result for many input slices. The matrices *X* and *S* consist of small integers (0,±1,±2,…), making it possible to realize them by a number of pre- and post-additions. In addition, in ConvNets multiple channels from the same layer can be computed at the same time. For example, a typical convolution layer with *C* channels will result in the following *C* output transforms *S*:

(12) $$s_{C}\left(p\right)=\sum_{c=1}^{C}S\left[{\left\{Ww\left(p\right)\right\}}_{c}⊙{\left\{Xx\left(p\right)\right\}}_{c}\right]$$

We can rewrite the equation as follows and only apply the output transform once *S* on the final sum. This amortizes the cost of the output transform over the number of channels in a layer.

(13) $$s_{C}\left(p\right)=S\sum_{c=1}^{C}\left[{\left\{Ww\left(p\right)\right\}}_{c}⊙{\left\{Xx\left(p\right)\right\}}_{c}\right]$$

Finally, the only dominant costs left over here are (L+N−1) elementwise multiplications from step 3.

### 4.5. A Fast Convolution Algorithm for Filtering of Dimension Three Using the Modified Toom–Cook Scheme

In our 1D-FALCON scheme, we have chosen an input block size of (6×1) to be convolved with (3×1) 1D filters. This results in a (4×1) block as output and we denote this algorithm as F(4×1,3×1,{6×1}). Alternatively, one can also start with a (4×1) input block and swap output and input transforms to obtain the same result as shown in Equation (34). Using this alternative approach we will now compute the necessary transformation matrices, namely, *W*, *X*, and *S*.

(14) $$w\left(p\right)=w_{0}+w_{1}p+w_{2}p^{2}$$

(15) $$x\left(p\right)=x_{0}+x_{1}p+x_{2}p^{2}+x_{3}p^{3}$$

(16) $$s\left(p\right)=w\left(p\right)x\left(p\right)=s_{0}+s_{1}p+s_{2}p^{2}+s_{3}p^{3}+s_{4}p^{4}+s_{5}p^{5}$$

since L=3 and N=4, L+N−3=4. Therefore we can choose $\beta_{0}=0,\beta_{1}=1,\beta_{2}=-1,\beta_{3}=2,$ and $\beta_{4}=-2.$ Now, let us calculate individual $w(\beta_{k})$ and $x(\beta_{k})$ as follows:

(17) $$\beta_{0}=0,\;\;w\left(\beta_{0}\right)=w_{0},\;\;x\left(\beta_{0}\right)=x_{0}$$

(18) $$\beta_{1}=1,\;\;w\left(\beta_{1}\right)=w_{0}+w_{1}+w_{2},\;\;x\left(\beta_{1}\right)=x_{0}+x_{1}+x_{2}+x_{3}$$

(19) $$\beta_{2}=-1,\;\;w\left(\beta_{2}\right)=w_{0}-w_{1}+w_{2},\;\;x\left(\beta_{2}\right)=x_{0}-x_{1}+x_{2}-x_{3}$$

(20) $$\beta_{3}=2,\;\;w\left(\beta_{3}\right)=w_{0}+2w_{1}+4w_{2},\;\;x\left(\beta_{3}\right)=x_{0}+2x_{1}+4x_{2}+8x_{3}$$

(21) $$\beta_{4}=-2,\;\;w\left(\beta_{4}\right)=w_{0}-2w_{1}+4w_{2},\;\;x\left(\beta_{3}\right)=x_{0}-2x_{1}+4x_{2}-8x_{3}$$

According to the modified Toom–Cook algorithm, the polynomial of degree (L+N−3) now can be expressed as follows:

(22) $$s^{{}^{\prime }}\left(\beta_{0}\right)=w\left(\beta_{0}\right)x\left(\beta_{0}\right)-w_{2}x_{3}\beta_{0}^{5}=w\left(\beta_{0}\right)x\left(\beta_{0}\right)$$

(23) $$s^{{}^{\prime }}\left(\beta_{1}\right)=w\left(\beta_{1}\right)x\left(\beta_{1}\right)-w_{2}x_{3}\beta_{1}^{5}=w\left(\beta_{1}\right)x\left(\beta_{1}\right)-w_{2}x_{3}$$

(24) $$s^{{}^{\prime }}\left(\beta_{2}\right)=w\left(\beta_{2}\right)x\left(\beta_{2}\right)-w_{2}x_{3}\beta_{2}^{5}=w\left(\beta_{2}\right)x\left(\beta_{2}\right)+w_{2}x_{3}$$

(25) $$s^{{}^{\prime }}\left(\beta_{3}\right)=w\left(\beta_{3}\right)x\left(\beta_{3}\right)-w_{2}x_{3}\beta_{3}^{5}=w\left(\beta_{3}\right)x\left(\beta_{3}\right)-32w_{2}x_{3}$$

(26) $$s^{{}^{\prime }}\left(\beta_{4}\right)=w\left(\beta_{4}\right)x\left(\beta_{4}\right)-w_{2}x_{3}\beta_{4}^{5}=w\left(\beta_{4}\right)x\left(\beta_{4}\right)+32w_{2}x_{3}$$

Using Lagrange interpolation

(27) $$\begin{array}{c} \;s^{{}^{\prime }}\left(p\right)=s^{{}^{\prime }}\left(\beta_{0}\right)\frac{\left(p-\beta_{1}\right)\left(p-\beta_{2}\right)\left(p-\beta_{3}\right)\left(p-\beta_{4}\right)}{\left(\beta_{0}-\beta_{1}\right)\left(\beta_{0}-\beta_{2}\right)\left(\beta_{0}-\beta_{3}\right)\left(\beta_{0}-\beta_{4}\right)}\\ +s^{{}^{\prime }}\left(\beta_{1}\right)\frac{\left(p-\beta_{0}\right)\left(p-\beta_{2}\right)\left(p-\beta_{3}\right)\left(p-\beta_{4}\right)}{\left(\beta_{1}-\beta_{0}\right)\left(\beta_{1}-\beta_{2}\right)\left(\beta_{1}-\beta_{3}\right)\left(\beta_{1}-\beta_{4}\right)}\\ +s^{{}^{\prime }}\left(\beta_{2}\right)\frac{\left(p-\beta_{0}\right)\left(p-\beta_{1}\right)\left(p-\beta_{3}\right)\left(p-\beta_{4}\right)}{\left(\beta_{2}-\beta_{0}\right)\left(\beta_{2}-\beta_{1}\right)\left(\beta_{2}-\beta_{3}\right)\left(\beta_{2}-\beta_{4}\right)}\\ +s^{{}^{\prime }}\left(\beta_{3}\right)\frac{\left(p-\beta_{0}\right)\left(p-\beta_{1}\right)\left(p-\beta_{2}\right)\left(p-\beta_{4}\right)}{\left(\beta_{3}-\beta_{0}\right)\left(\beta_{3}-\beta_{1}\right)\left(\beta_{3}-\beta_{2}\right)\left(\beta_{3}-\beta_{4}\right)}\\ +s^{{}^{\prime }}\left(\beta_{4}\right)\frac{\left(p-\beta_{0}\right)\left(p-\beta_{1}\right)\left(p-\beta_{2}\right)\left(p-\beta_{3}\right)}{\left(\beta_{4}-\beta_{0}\right)\left(\beta_{4}-\beta_{1}\right)\left(\beta_{4}-\beta_{2}\right)\left(\beta_{4}-\beta_{3}\right)} \end{array}$$

the above equation can be simplified further and can be re-arranged in the polynomial form as follows:

(28) $$\begin{array}{c} \;s^{{}^{\prime }}\left(p\right)=s^{{}^{\prime }}\left(\beta_{0}\right)+p\left(\frac{4}{6}s^{{}^{\prime }}\left(\beta_{1}\right)-\frac{4}{6}s^{{}^{\prime }}\left(\beta_{2}\right)-\frac{2}{24}s^{{}^{\prime }}\left(\beta_{3}\right)+\frac{2}{24}s^{{}^{\prime }}\left(\beta_{4}\right)\right)\\ +p^{2}\left(-\frac{5}{4}s^{{}^{\prime }}\left(\beta_{0}\right)+\frac{4}{6}s^{{}^{\prime }}\left(\beta_{1}\right)+\frac{4}{6}s^{{}^{\prime }}\left(\beta_{2}\right)-\frac{1}{24}s^{{}^{\prime }}\left(\beta_{3}\right)-\frac{1}{24}s^{{}^{\prime }}\left(\beta_{4}\right)\right)\\ +p^{3}\left(-\frac{1}{6}s^{{}^{\prime }}\left(\beta_{1}\right)+\frac{1}{6}s^{{}^{\prime }}\left(\beta_{2}\right)+\frac{2}{24}s^{{}^{\prime }}\left(\beta_{3}\right)-\frac{2}{24}s^{{}^{\prime }}\left(\beta_{4}\right)\right)\\ +p^{4}\left(\frac{1}{4}s^{{}^{\prime }}\left(\beta_{0}\right)-\frac{1}{6}s^{{}^{\prime }}\left(\beta_{1}\right)-\frac{1}{6}s^{{}^{\prime }}\left(\beta_{2}\right)+\frac{1}{24}s^{{}^{\prime }}\left(\beta_{3}\right)+\frac{1}{24}s^{{}^{\prime }}\left(\beta_{4}\right)\right) \end{array}$$

Since we have modified Toom–Cook algorithm to reduce number of additions, we can get back s(p) by using

(29) $$s\left(p\right)=s^{{}^{\prime }}\left(p\right)+w_{2}x_{3}p^{5}$$

Finally, we have the output in matrix form by replacing all $\beta_{k}$ in the previous equation,

(30) $$\left[\begin{array}{c} s_{0}\\ s_{1}\\ s_{2}\\ s_{3}\\ s_{4}\\ s_{5} \end{array}\right]=\left[\begin{array}{cccccc} 4 & 0 & 0 & 0 & 0 & 0\\ 0 & 4 & -4 & -2 & 2 & 4\\ -5 & 4 & 4 & -1 & -1 & 0\\ 0 & -1 & 1 & 2 & -2 & -5\\ 1 & -1 & -1 & 1 & 1 & 0\\ 0 & 0 & 0 & 0 & 0 & 1 \end{array}\right]\left[\begin{array}{cccccc} W_{0} & & & & & \\ & W_{1} & & & & \\ & & W_{2} & & & \\ & & & W_{3} & & \\ & & & & W_{4} & \\ & & & & & W_{5} \end{array}\right]\left[\begin{array}{cccc} 1 & 0 & 0 & 0\\ 1 & 1 & 1 & 1\\ 1 & -1 & 1 & -1\\ 1 & 2 & 4 & 8\\ 1 & -2 & 4 & -8\\ 0 & 0 & 0 & 1 \end{array}\right]\left[\begin{array}{c} x_{0}\\ x_{1}\\ x_{2}\\ x_{3} \end{array}\right]$$

where

(31) $$\left[\begin{array}{c} W_{0}\\ W_{1}\\ W_{2}\\ W_{3}\\ W_{4}\\ W_{5} \end{array}\right]=\left[\begin{array}{ccc} \frac{1}{4} & 0 & 0\\ \frac{1}{6} & \frac{1}{6} & \frac{1}{6}\\ \frac{1}{6} & -\frac{1}{6} & \frac{1}{6}\\ \frac{1}{24} & \frac{1}{12} & \frac{1}{6}\\ \frac{1}{24} & -\frac{1}{12} & \frac{1}{6}\\ 0 & 0 & 1 \end{array}\right]\left[\begin{array}{c} w_{0}\\ w_{1}\\ w_{2} \end{array}\right]$$

The Toom–Cook algorithm can be viewed as a method of factoring matrices and can be expressed as the following form (⊙ denotes element-wise multiplication):

(32) s=X[(Ww)⊙(Sx)]

We can transpose this solution for a larger block size using matrix exchange theorem from linear algebra. According to matrix exchange theorem, if we have a matrix M which can be factored as:

(33) s=XDS

where *D* is a diagonal matrix, then it can also be factored as:

(34) $$s={\left(\bar{S}\right)}^{T}D{\left(\underline{X}\right)}^{T}$$

where $\bar{S}$ is the matrix obtained from *S* by reversing the order of its columns, and $\underline{X}$ is the matrix obtained from *X* by reversing the order of its rows. We can now apply the same on our final equation and have an alternative form as follows:

(35) $$s=S^{T}\left[\left(Ww\right)⊙\left(X^{T}x\right)\right]$$

Finally, we obtain the transformation matrices $S^{T}$, $X^{T}$, and *W* from Equations (36)–(38) respectively.

(36) $$\left[\begin{array}{c} s_{0}\\ s_{1}\\ s_{2}\\ s_{3} \end{array}\right]=\left[\begin{array}{cccccc} 1 & 1 & 1 & 1 & 1 & 0\\ 0 & 1 & -1 & 2 & -2 & 0\\ 0 & 1 & 1 & 4 & 4 & 0\\ 0 & 1 & -1 & 8 & -8 & 1 \end{array}\right]\left[\begin{array}{c} S_{0}\\ S_{1}\\ S_{2}\\ S_{3}\\ S_{4}\\ S_{5} \end{array}\right]$$

where

(37) $$\left[\begin{array}{c} S_{0}\\ S_{1}\\ S_{2}\\ S_{3}\\ S_{4}\\ S_{5} \end{array}\right]=\left[\begin{array}{cccccc} W_{0} & & & & & \\ & W_{1} & & & & \\ & & W_{2} & & & \\ & & & W_{3} & & \\ & & & & W_{4} & \\ & & & & & W_{5} \end{array}\right]⊙\left[\begin{array}{cccccc} 4 & 0 & -5 & 0 & 1 & 0\\ 0 & -4 & -4 & 1 & -1 & 0\\ 0 & 4 & -4 & -1 & -1 & 0\\ 0 & -2 & -1 & 2 & 1 & 0\\ 0 & 2 & -1 & -2 & 1 & 0\\ 0 & 4 & 0 & -5 & 0 & 1 \end{array}\right]\left[\begin{array}{c} x_{0}\\ x_{1}\\ x_{2}\\ x_{3}\\ x_{4}\\ x_{6} \end{array}\right]$$

where

(38) $$\left[\begin{array}{c} W_{0}\\ W_{1}\\ W_{2}\\ W_{3}\\ W_{4}\\ W_{5} \end{array}\right]=\left[\begin{array}{ccc} \frac{1}{4} & 0 & 0\\ \frac{1}{6} & \frac{1}{6} & \frac{1}{6}\\ \frac{1}{6} & -\frac{1}{6} & \frac{1}{6}\\ \frac{1}{24} & \frac{1}{12} & \frac{1}{6}\\ \frac{1}{24} & -\frac{1}{12} & \frac{1}{6}\\ 0 & 0 & 1 \end{array}\right]\left[\begin{array}{c} w_{0}\\ w_{1}\\ w_{2} \end{array}\right]$$

## 5. Results and Discussion

In order to evaluate the effectiveness of our scheme we compared it against several popular networks targeting the MNIST, CIFAR-10, ImageNet, and PASCAL VOC datasets. In this paper, we demonstrate our result for the VGG-16 model, which won the the ImageNet challenge in 2014 [30]. VGG-16 is a deep architecture and consists of 13 convolutional layers out of a total of16 layers. To make a comparison with a wide variety of speedup techniques, we chose a direct 2D convolutional scheme [30], a low-rank scheme based on the Tucker decomposition [31], two popular pruning techniques ([7,32]), a sparsification scheme [33], and the 2D Winograd filtering scheme [23].

We used three main metrics for comparison:

- **MULs:** Total number of strong operations (i.e., multiplications) in the convolutional layers
- **Speedup:** Total speedup achieved as compared to baseline 2D convolution
- **Fine-Tuning Time:** Average fine-tuning time in number of epochs. The fine-tuning is the process of re-training a CNN after having trained it once and then having reduced its complexity. An epoch is a complete pass through the training set.

As can be seen from Table 1, our 1D-FALCON scheme achieves significant speedup compared to other schemes and does not require a long fine-tuning time. The overall speedup comes from combined application of both the low-rank approximation scheme and the fast 1D convolution technique using the modified Toom–Cook algorithm. The following section highlights the detail speed up achieved from the individual stages of our optimization pipeline.

### 5.1. Speedup from the Low-Rank Approximation Stage: 

**Figure 6:**
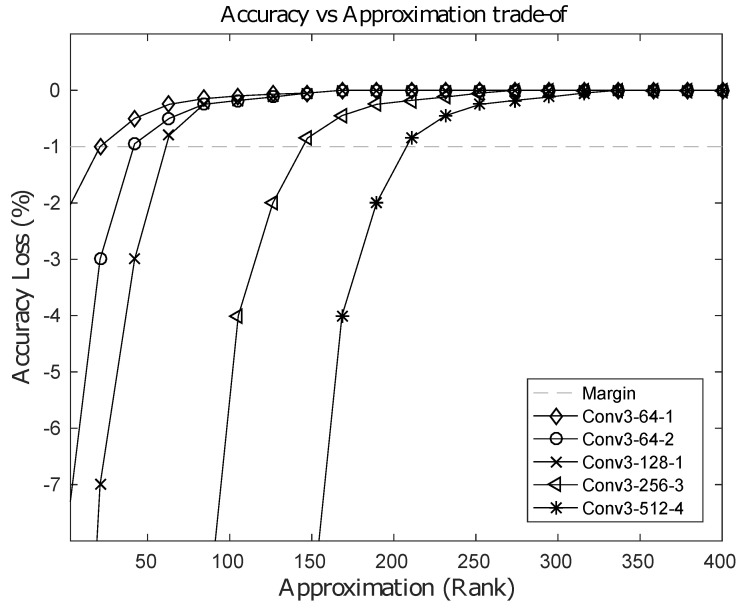
Loss in accuracy(%) vs. rank-approximation of selected layers from the VGG16 Model.

The computational cost of the baseline 2D direct convolution is $O(F_{I}MNmnF_{O})$, where each input feature map is of size (M×N), spatial two-dimensional kernels are of size (m×n) and $F_{I}$, $F_{O}$ are the input and output channels within a layer, respectively. However, using our 1D-FALCON approximation scheme, the computational costs for the vertical stage and the horizontal stage are $O(F_{I}MNmR)$, $O(RMNnF_{O})$, respectively, resulting in a total computational cost of $O(\left(mF_{I}+nF_{O}\right)MNR)$. If we choose *R* such that $R\left(mF_{I}+nF_{O}\right)\;<<\;mn\left(F_{I}F_{O}\right)$, then the computational cost can be reduced. In practice, current state-of-the-art convolutional neural networks use square kernels. Hence, let us assume m=n=p, which is the size of the kernel in the model. Using this assumption, the condition can be simplified to $R\left(F_{I}+F_{O}\right)\;<<\;pF_{I}F_{O}$. In addition, most modern ConvNets use more channels in the higher layers than the corresponding lower layers, i.e., the channel ratio $\frac{F_{O}}{F_{I}}\;>>\;1$. The higher the ratio, the larger the value of *R* can be. In most layers, the computation cost can be reduced by *p*, which is the dimension of the kernel in the respective layer. Our evaluation on VGG-16 showed an average speedup of 3–5 times in all layers and a maximum speedup of 8–9 times on many individual layers. Table 2 shows the layer-wise speedup of convolutions achieved in the VGG-16 model using an Intel i7-5930k system. It is possible to push the limit of approximation further with an increased loss in classification accuracy. This increased amount of loss can be recovered back by fine-tuning the model; however, more approximation in the layer leads to longer fine-tuning time. After a certain limit in the rank, the original baseline accuracy cannot be recovered back to an acceptable level. Figure 6 shows an accuracy vs. approximation trade-off for few selected layers from the VGG-16 model. In the figure the horizontal dashed line represents an acceptable loss of accuracy of 1% from baseline.

### 5.2. Speedup from the Fast Convolution Stage 

The 1D Toom–Cook algorithm requires (N+L−1) multiplications compared to a direct implementation which will require (N×L) multiplications, where N, L are the dimensions of an input feature slice and a 1D filter, respectively. In case of VGG-16 model, we chose N=4 and L=3, resulting in 2× savings in the total number of multiplications. As our modified VGG-16 model has vertical and horizontal stages, in total it achieves 2× savings in the number of multiplications in each 1D stage. A 4× reduction in computational intensity is also possible if we use a variant of the algorithm using output block size of 6. The $S^{T}$, $X^{T}$ and *W* transformation matrices corresponding to this variant is shown in Appendix D. However, this speedup is achievable at the cost of a seven-fold increase in the memory footprint of the filters.

### 5.3. Efficient Use of Memory Bandwidth and Improved Local Reuse

Our 1D-FALCON scheme not only helps in reducing overall computational intensity but also reduces cost of storage that arises from the convolutional layers. The cost of storage without application of this scheme is $F_{I}F_{O}p^{2}$, whereas cost reduces to $\left(F_{I}pR+RpF_{O}\right)$ after approximation and separating the kernels into two rectangular ones. If we choose $R\;<<\;p\left(F_{I}F_{O}\right)/\left(F_{I}+F_{O}\right)$, significant savings can be made for the storage costs of the kernels. Table 3 shows an average 5× reduction in the overall memory footprint of the model, whereas many individual layers achieve a 9–10× reduction. Fetching data from off-chip main memory (DRAM) generates costs an order of magnitude greater than from on-chip or local storage [34,35]. Chen et al. in their Eyeriss research project showed that row-stationary 1D convolution is the optimal solution for throughput and energy efficiency, as compared to the scheme that uses classical 2D convolution [36]. Separable filters enable row-stationary 1D convolutions by reducing the number of unnecessary data loads in padded convolution, dividing the convolution into two 1D stages. To preserve information, many convolutional networks use zero-padding in many layers. Around the image tile, there is an *apron* of pixels that is required in order to filter the image tile. Note that the *apron* of one block also overlaps with the adjacent blocks. If we separate the convolution into vertical and horizontal passes, it is no longer necessary to load the top and bottom *apron* regions for the horizontal stage of computation. Similarly, for the vertical stage, it is no longer necessary to load the left and right *apron* regions . This allows more efficient use of the available memory bandwidth and on-chip storage. In case of strided convolution, this approach works very well.

### 5.4. Extension of the 1D Algorithm to a 2D Variant and Its Limitations

We can extend the one-dimensional convolution solution shown in Equation (35) for two-dimensional convolution easily by nesting the first transforms inside the second transforms as follows:

(39) $$s=S^{T}\left[\left(WwW^{T}\right)⊙\left(X^{T}xX\right)\right]S$$

**Figure 7:**
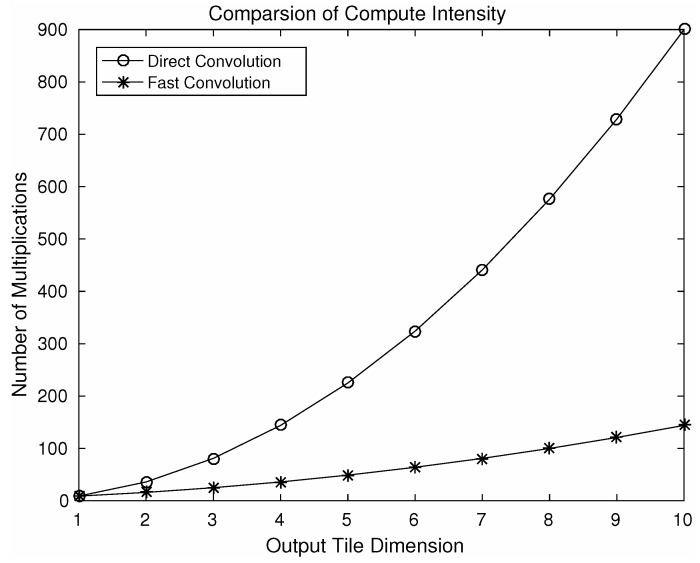
Comparison of computational intensity using a variation of the algorithm F(M×M, 3×3), where M denotes output tile dimension.

**Figure 8:**
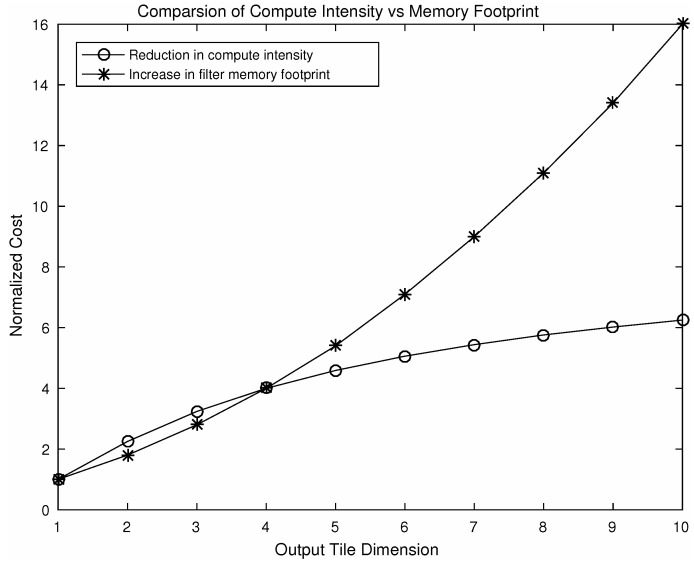
Comparison of reduction in computational intensity and the increase in filter memory footprint using a variation of the algorithm F(M×M, 3×3) where M denotes output tile dimension.

As a result of nesting, in a 2D convolution ${\left(L+N-1\right)}^{2}$, element-wise multiplications will be required. By choosing different values of *N* a number of variants of this algorithm can be produced using the steps shown in Figure 5. Using different variants of the algorithm, speeding up can be achieved. Figure 7 shows a comparison of computational intensity between fast convolution and direct convolution for different choices of output tile size. We can see from the figure that larger tile sizes lead to a higher speedup. However, for the larger tile size the associated cost of memory footprint increases dramatically. As we choose larger tile sizes, the filter tile size also needs to be increased to match the dimensions, which results in an increased memory footprint. A comparison between reduction in computational intensity and the increase in memory footprint associated with filters is shown in Figure 8. As an example, using an output tile size of 6, the computational intensity can be reduced by almost five times. However this speed up will result in a seven-fold increase in memory footprint associated with the filters, which may be significant for embedded systems as they do not have large on-chip memory.

## 6. Conclusions

In this paper, we demonstrated that co-optimization of internal structure of the ConvNet models and the underlying implementation of the fundamental algebraic operation form an efficient approach to speedup inference in convolutional neural networks. In the first stage of our optimization pipeline, to facilitate the structural optimization of the models we introduced an easy-to-implement and a correlation-based mathematically well-grounded technique which approximates each filter bank by exploiting inherent redundancies among feature maps in the ConvNets. Unlike many iterative pruning and regularization techniques, our scheme does not require any time consuming fine-tuning and yet preserves the baseline accuracy. In addition, the availability of several pre-tunes models with different performance–accuracy targets can provide significant advantages for deploying ConvNets on fast time-to-market emerging applications. The first stage of reduction in computational intensity is augmented with a further fast convolution stage using the modified Toom–Cook algorithm. In this stage, the total number of strong operations is reduced dramatically without any approximations that may affect accuracy. We have evaluated our 1D-FALCON optimization scheme on a variety of ConvNets targeting different datasets and model sizes. The results from the evaluation running on a range of real hardware provides strong evidence that a significant speedup in ConvNet can be achieved without sacrificing baseline accuracy by jointly optimizing the structure of the network and the underlying implementation of fundamental convolution operations.

## Figures and Tables

**Table 1:** A comparison of speedup of VGG-16 using different schemes.

Optimization Scheme	#MULs	Speedup	Top-5 Error (%)	Fine-Tuning Time
2D Convolution [30]	15.3G	1.0×	9.4	None
Group-Wise Sparsification [33]	7.6G	2.0×	10.1	>10 epochs
Iterative Pruning [32]	4.5G	3.4×	13.0	60 epochs
Winograd’s Filtering [23]	3.8G	4.0×	9.4	None
Pruning+Retraining [7]	3.0G	5.0×	10.88	20–40 epochs
Tucker Decomposition [31]	3.0G	5.0×	11.60	5–10 epochs
1D FALCON [Our scheme]	1.3G	11.4×	9.5	1–2 epochs

**Table 2:** VGG16 layerwise speedup of convolution on i7-5930k (per image) with no loss in the baseline accuracy of 90.5%.

Layer	Original (ms)	Compressed (ms)	Speedup
conv3-64-1.1	28.5	15.0	1.9×
conv3-64-1.2	168.1	32.3	5.2×
conv3-128-2.1	74.1	25.6	2.9×
conv3-128-2.2	147.3	42.1	3.5×
conv3-256-3.1	67.3	15.7	4.3×
conv3-256-3.2	134.2	25.8	5.2×
conv3-256-3.3	134.5	27.4	4.9×
conv3-512-4.1	65.2	12.8	5.1×
conv3-512-4.2	129.9	22.0	5.9×
conv3-512-4.3	130.1	21.3	6.1×
conv3-512-5.1	33.4	4.3	7.8×
conv3-512-5.2	33.5	4.2	7.9×
conv3-512-5.3	33.4	4.2	7.9×
Total	1432.3	252.8	5.7×

**Table 3:** VGG16 model approximation summary.

Layer	No. of Parameters	Compressed Column $\left(F_{I}\times m\times n\times V_{R}\right)$	Compressed Row $\left(V_{R}\times m\times n\times F_{O}\right)$	Reduction in Layer Size
conv3x3-64-1.1	2K	3×3×1×4	4×1×3×64	2.1×
conv3x3-64-1.2	37K	64×3×1×12	12×1×3×64	8.0×
conv3x3-128-2.1	74K	64×3×1×40	40×1×3×128	3.2×
conv3x3-128-2.2	148K	128×3×1×40	40×1×3×128	4.8×
conv3x3-256-3.1	295K	128×3×1×50	50×1×3×256	5.1×
conv3x3-256-3.2	590K	256×3×1×60	60×1×3×256	6.4×
conv3x3-256-3.3	590K	256×3×1×70	70×1×3×256	5.5×
conv3x3-512-4.1	1M	512×3×1×80	80×1×3×512	6.4×
conv3x3-512-4.2	2M	512×3×1×100	100×1×3×512	7.7×
conv3x3-512-4.3	2M	512×3×1×110	110×1×3×512	7.0×
conv3x3-512-5.1	2M	512×3×1×80	80×1×3×512	9.6×
conv3x3-512-5.2	2M	512×3×1×78	78×1×3×512	9.8×
conv3x3-512-5.3	2M	512×3×1×78	78×1×3×512	9.8×

## Abbreviations

The following abbreviations are used in this manuscript:

ConvNet	Convolutional Neural Network
ILSVRC	The ImageNet Large Scale Visual Recognition Challenge
1D-FALCON	One-Dimensional Fast Approximate Low-rank Convolution

## Appendix A. Algorithm F(2×1, 3×1, {4×1})

The following 1D algorithm can be used to convolve a (4×1) input with a (3×1) filter. The output of this algorithm will produce a (2×1) output block. This 1D algorithm can also be easily nested for use with a (3×3) filter on a (4×4) input tile to produce a (2×2) output region. The output transformation can be computed as follows:

(A1) $$\left[\begin{array}{c} s_{0}\\ s_{1} \end{array}\right]=\left[\begin{array}{cccc} 1 & 1 & 1 & 0\\ 0 & 1 & -1 & 1 \end{array}\right]\left[\begin{array}{c} S_{0}\\ S_{1}\\ S_{2}\\ S_{3} \end{array}\right]$$

where intermediate results can be computed as follows:

(A2) $$\left[\begin{array}{c} S_{0}\\ S_{1}\\ S_{2}\\ S_{3} \end{array}\right]=\left[\begin{array}{cccc} W_{0} & & & \\ & W_{1} & & \\ & & W_{2} & \\ & & & W_{3} \end{array}\right]⊙\left[\begin{array}{cccc} 1 & 0 & -1 & 0\\ 0 & 1 & 1 & 0\\ 0 & -1 & 1 & 0\\ 0 & -1 & 0 & 1 \end{array}\right]\left[\begin{array}{c} x_{0}\\ x_{1}\\ x_{2}\\ x_{3} \end{array}\right]$$

and the filter transformation can be obtained as follows:

(A3) $$\left[\begin{array}{c} W_{0}\\ W_{1}\\ W_{2}\\ W_{3} \end{array}\right]=\left[\begin{array}{ccc} 1 & 0 & 0\\ \frac{1}{2} & \frac{1}{2} & \frac{1}{2}\\ \frac{1}{2} & -\frac{1}{2} & \frac{1}{2}\\ 0 & 0 & 1 \end{array}\right]\left[\begin{array}{c} w_{0}\\ w_{1}\\ w_{2} \end{array}\right]$$

## Appendix B. Algorithm F(3×1, 3×1, {5×1})

The following 1D algorithm can be used to convolve a (5×1) input with a (3×1) filter. The output of this algorithm will produce a (3×1) output block. This 1D algorithm can also be easily nested to be used with a (3×3) filter on a (5×5) input tile to produce a (3×3) output region. The output transformation can be computed as follows:

(A4) $$\left[\begin{array}{c} s_{0}\\ s_{1}\\ s_{2} \end{array}\right]=\left[\begin{array}{ccccc} 1 & 1 & 1 & 1 & 0\\ 0 & 1 & -1 & 2 & 0\\ 0 & 1 & 1 & 4 & 1 \end{array}\right]\left[\begin{array}{c} S_{0}\\ S_{1}\\ S_{2}\\ S_{3}\\ S_{4} \end{array}\right]$$

where intermediate results can be computed as follows:

(A5) $$\left[\begin{array}{c} S_{0}\\ S_{1}\\ S_{2}\\ S_{3}\\ S_{4} \end{array}\right]=\left[\begin{array}{ccccc} W_{0} & & & & \\ & W_{1} & & & \\ & & W_{2} & & \\ & & & W_{3} & \\ & & & & W_{4} \end{array}\right]⊙\left[\begin{array}{ccccc} 2 & -1 & -2 & 1 & 0\\ 0 & -2 & -1 & 1 & 0\\ 0 & 2 & -3 & 1 & 0\\ 0 & -1 & 0 & 1 & 0\\ 0 & 2 & -1 & -2 & 1 \end{array}\right]\left[\begin{array}{c} x_{0}\\ x_{1}\\ x_{2}\\ x_{3}\\ x_{4} \end{array}\right]$$

and the filter transformation can be obtained as follows:

(A6) $$\left[\begin{array}{c} W_{0}\\ W_{1}\\ W_{2}\\ W_{3}\\ W_{4} \end{array}\right]=\left[\begin{array}{ccc} \frac{1}{2} & 0 & 0\\ -\frac{1}{2} & -\frac{1}{2} & -\frac{1}{2}\\ -\frac{1}{6} & \frac{1}{6} & -\frac{1}{6}\\ \frac{1}{6} & \frac{1}{3} & \frac{2}{3}\\ 0 & 0 & 1 \end{array}\right]\left[\begin{array}{c} w_{0}\\ w_{1}\\ w_{2} \end{array}\right]$$

## Appendix C. Additional Results from Other Widely Used CNNs

We have applied our technique to many other widely used CNNs trained on the ImageNet dataset. In this section we provide a comprehensive summary of the speedup achieved from our experiments.

**Table A1:** A comparison of speedup of VGG-16 using different schemes.

CNNs	#MULs (Millions)	Speed-Up	Top-5 Error (%)	Fine-Tuning Time
AlexNet [37]	692	12.1×	19.8	1 epoch
VGG-16 [30]	15,300	11.4×	9.5	1–2 epochs
Inception-v1 [38]	1428	7.2×	10.7	3 epochs
ResNet-152 [39]	11,300	6.2×	5.3	2–3 epochs

## Appendix D. Algorithm F(6×1, 3×1, {8×1})

The following 1D algorithm can be used to convolve a (8×1) input with a (3×1) filter. The output of this algorithm will produce a (6×1) output block. This 1D algorithm can also be easily nested to be used with a (3×3) filter on a (8×8) input tile to produce a (6×6) output region. The output transformation can be computed as follows:

(A7) $$\left[\begin{array}{c} s_{0}\\ s_{1}\\ s_{2}\\ s_{3}\\ s_{4}\\ s_{5} \end{array}\right]=\left[\begin{array}{cccccccc} 1 & 1 & 1 & 1 & 1 & 1 & 1 & 0\\ 0 & 1 & -1 & 2 & -2 & 3 & -3 & 0\\ 0 & 1 & 1 & 4 & 4 & 9 & 9 & 0\\ 0 & 1 & -1 & 8 & -8 & 27 & -27 & 0\\ 0 & 1 & 1 & 16 & 16 & 81 & 81 & 0\\ 0 & 1 & -4 & 32 & -32 & 243 & -243 & 1 \end{array}\right]\left[\begin{array}{c} S_{0}\\ S_{1}\\ S_{2}\\ S_{3}\\ S_{4}\\ S_{5}\\ S_{6}\\ S_{7} \end{array}\right]$$

where intermediate results can be computed as follows:

(A8) $$\left[\begin{array}{c} S_{0}\\ S_{1}\\ S_{2}\\ S_{3}\\ S_{4}\\ S_{5}\\ S_{6}\\ S_{7} \end{array}\right]=\left[\begin{array}{cccccccc} W_{0} & & & & & & & \\ & W_{1} & & & & & & \\ & & W_{2} & & & & & \\ & & & W_{3} & & & & \\ & & & & W_{4} & & & \\ & & & & & W_{5} & & \\ & & & & & & W_{6} & \\ & & & & & & & W_{7} \end{array}\right]⊙X^{T}\left[\begin{array}{c} x_{0}\\ x_{1}\\ x_{2}\\ x_{3}\\ x_{4}\\ x_{5}\\ x_{6}\\ x_{7} \end{array}\right]$$

where

(A9) $$X^{T}=\left[\begin{array}{cccccccc} 36 & 0 & -49 & 0 & 14 & 0 & -1 & 0\\ 0 & 36 & 36 & -13 & -13 & 1 & 1 & 0\\ 0 & -36 & 36 & 13 & -13 & -1 & 1 & 0\\ 0 & 18 & 9 & -20 & -10 & 2 & 1 & 0\\ 0 & -18 & 9 & 20 & -10 & -2 & 1 & 0\\ 0 & 12 & 4 & -15 & -5 & 3 & 1 & 0\\ 0 & -12 & 4 & 15 & -5 & -3 & 1 & 0\\ 0 & -36 & 0 & 49 & 0 & -14 & 0 & 1 \end{array}\right]$$

and the filter transformation can be obtained as follows:

(A10) $$\left[\begin{array}{c} W_{0}\\ W_{1}\\ W_{2}\\ W_{3}\\ W_{4}\\ W_{5}\\ W_{6}\\ W_{7} \end{array}\right]=\left[\begin{array}{ccc} \frac{1}{36} & 0 & 0\\ \frac{1}{48} & \frac{1}{48} & \frac{1}{48}\\ \frac{1}{48} & -\frac{1}{48} & \frac{1}{48}\\ -\frac{1}{120} & -\frac{1}{60} & -\frac{1}{30}\\ -\frac{1}{120} & \frac{1}{60} & -\frac{1}{30}\\ \frac{1}{720} & \frac{1}{240} & \frac{1}{80}\\ \frac{1}{720} & -\frac{1}{240} & \frac{1}{80}\\ 0 & 0 & 1 \end{array}\right]\left[\begin{array}{c} w_{0}\\ w_{1}\\ w_{2} \end{array}\right]$$
